# The Interaction Between CitMYB52 and CitbHLH2 Negatively Regulates Citrate Accumulation by Activating *CitALMT* in Citrus Fruit

**DOI:** 10.3389/fpls.2022.848869

**Published:** 2022-03-21

**Authors:** Shengchao Liu, Xincheng Liu, Bangrui Gou, Dengliang Wang, Chunrong Liu, Jun Sun, Xueren Yin, Donald Grierson, Shaojia Li, Kunsong Chen

**Affiliations:** ^1^College of Agriculture and Biotechnology, Zhejiang University, Hangzhou, China; ^2^Zhejiang Provincial Key Laboratory of Horticultural Plant Integrative Biology, Zhejiang University, Hangzhou, China; ^3^Quzhou Academy of Agricultural Science, Quzhou, China; ^4^Zhejiang Agricultural Technology Extension Center, Hangzhou, China; ^5^The State Agriculture Ministry Laboratory of Horticultural Plant Growth, Development and Quality Improvement, Zhejiang University, Hangzhou, China; ^6^Division of Plant and Crop Sciences, School of Biosciences, University of Nottingham, Loughborough, United Kingdom

**Keywords:** citrus fruit, citric acid, aluminum-activated malate transporter, transcriptional regulation, MYB-bHLH protein complex

## Abstract

Citric acid plays significant roles in numerous physiological processes in plants, including carbon metabolism, signal transduction, and tolerance to environmental stress. For fruits, it has a major effect on fruit organoleptic quality by directly influencing consumer taste. Citric acid in citrus is mainly regulated by the balance between synthesis, degradation, and vacuolar storage. The genetic and molecular regulations of citric acid synthesis and degradation have been comprehensively elucidated. However, the transporters for citric acid in fruits are less well understood. Here, an aluminum-activated malate transporter, CitALMT, was characterized. Transient overexpression and stable transformation of *CitALMT* significantly reduced citrate concentration in citrus fruits and transgenic callus. Correspondingly, transient RNA interference-induced silencing of *CitALMT* and increased citrate significantly, indicating that CitALMT plays an important role in regulating citrate concentration in citrus fruits. In addition, dual-luciferase assays indicated that CitMYB52 and CitbHLH2 could trans-activate the promoter of *CitALMT*. EMSA analysis showed that CitbHLH2 could physically interact with the E-box motif in the *CitALMT* promoter. Bimolecular fluorescence complementation assays, yeast two-hybrid, coimmunoprecipitation and transient overexpression, and RNAi assay indicated that the interaction between CitMYB52 and CitbHLH2 could synergistically trans-activate *CitALMT* to negatively regulate citrate accumulation.

## Introduction

In plants, organic acids are of fundamental importance and play significant roles in numerous physiological processes. They serve as respiratory substrates in the tricarboxylic acid (TCA) cycle, precursors for numerous primary metabolites, such as amino acids, fatty acids, and secondary metabolites, such as aromatics and terpenoids (Ladanyia, 2008). Changes in organic acids levels can be perceived and have a major impact on the abundance of nucleus-encoded transcripts involved in photosynthesis, protein synthesis, biotic and abiotic stress (Finkemeier et al., 2013; Dyson et al., 2016). In addition, organic acids are involved in chemical modification of proteins, such as lysine succinylation (Zhang et al., 2011; Weinert et al., 2013), and play a role in the hygroscopicity of inorganic salts, such as ammonium sulfate, sodium chloride, and nitrate (Wang et al., 2017, 2018 Wu et al., 2018).

Citrus is the world’s most cultivated fruit and citric acid is the most important organic acid in citrus fruit, which serves as a typical material for citric acid metabolism studies. Agronomic factors (temperature, water, light, mineral elements, etc.) greatly affect citrate concentrate during fruit development and postharvest stage. In “Ponkan” fruit, hot air treatment could effectively reduce citric acid concentration in postharvest citrus (Li et al., 2021). Iron deficiency induced a decrease in cytoplasmic aconitase and a concomitant increase in citrate levels (Shlizerman et al., 2007). However, intrinsic genetic characteristics determine the citric acid concentration in different citrus varieties and fruit development periods. In recent years, the genetic and molecular regulations of citric acid synthesis and degradation have been widely studied. Citrate concentration in fruit cells is codetermined by the balance between synthesis, degradation, and vacuolar storage (Zampini et al., 2008; Wang et al., 2014). In *Malus xiaojinensis*, when *MxCS2* was introduced into *Arabidopsis*, citrate synthase was enhanced and citrate concentration was increased (Han et al., 2015). Genomic and transcriptomic analyses of wild mandarin populations and abundant mandarin varieties indicated that *CitAco2* and *CitAco3* are associated with lower citrate concentration (Wang et al., 2018), transient overexpression of *CitAco3* in citrus leaves and fruits could significantly decrease the citrate concentration (Li et al., 2017).

In mature citrus fruits, most of the citric acid is stored in vacuoles and contributes to flavor, nutrition, and juice quality. Thus, vacuolar transport processes may play a major role in determining citric acid accumulation (Etienne et al., 2013). The transport of citrate is related to the electrochemical gradient and transport carriers across the tonoplast (Huang et al., 2021). Proton pumps cause a massive proton influx that generates an acidic vacuolar pH and electrochemical gradient, which provides a strong driving force for citrate transportation across the tonoplast. An H^+^-ATPase gene *CsPH8* may regulate the accumulation of citric acid in citrus (Shi et al., 2015). Subsequent studies showed that the low acidity of citrus fruit is directly related to the downregulation of proton pump genes *CitP*H1 and *CitPH5*, which is associated with mutations that disrupt expression of MYB, HLH, and/or WRKY transcription factors homologous to those activating PH1 and PH5 in petunia (Strazzer et al., 2019). In addition, the transport of citrate is achieved by the manipulation of anion channels or transport carriers on the tonoplast (Huang et al., 2021). CsCit1 may act as a vacuolar citrate/H^+^ symporter in *Citrus* (Shimada et al., 2006), but the homolog in *Arabidopsis*, AttDT, was shown not to be the main tonoplast citrate carrier (Hurth et al., 2005). In citrus fruit, the major transport carriers of citric acid are poorly understood.

In the present study, an aluminum-activated malate transporter CitALMT was identified and functionally characterized by transient overexpression and RNA interference-induced silencing (RNAi) in citrus fruits and stable transgenic callus. Two transcription factors, CitMYB52 (Ciclev10001803m) and CitbHLH2 (Ciclev10005233m), trans-activated the *CitALMT* promoter. CitbHLH2 directly interacted with the E-box motif in the *CitALMT* promoter. Yeast two-hybrid assay, BiFC assay, and CoIP assay indicated that CitMYB52 and CitbHLH2 could interact with each other physically. Further transient dual overexpression and RNAi of *CitMYB52* and C*itbHLH2* in citrus fruits validated the role of this interaction in citrate accumulation by regulating transcript level of *CitALMT*. These findings provide new insights into the molecular basis of citrate modulation in citrus fruits.

## Materials and Methods

### Plant Materials

“Ponkan” (Citrus *reticulata* Blanco cv. Ponkan) fruits were obtained from a commercial orchard in Quzhou (Zhejiang, China) at different developmental stages, 120, 135, 150, 165, and 180 days after full bloom (DAFB) in 2017. At each sampling point, the size and appearance of the collected fruits from three different trees for three biological replicates are uniform. The flesh samples were taken and frozen in liquid nitrogen and stored at −80°C for subsequent experiments. Citrus hot air treatment, “Ordinary Ponkan (OPK)” and its spontaneous early maturing mutant “Zaoshu Ponkan (ZPK)” were described in detail in our previous study (Li et al., 2016, 2021).

### Transcriptome Data Analysis

In the two previous reports, the genetic and molecular regulation of citric acid synthesis and degradation have been studied in high- and low-citrate containing fruits. The fruit flesh samples of “Gaocheng” (“GC”) and “Satsuma mandarin” (“SM”) were used for transcriptomic analysis to explore the inner mechanism of difference in acidity among different varieties (Lin et al., 2015a); the fruit flesh samples of different developmental stages of “Ponkan” (“PK”) fruits were used to study the relationship between genes and metabolites during fruit development (Lin et al., 2015b). Using RNA-seq results from our previous reports, we obtained the DEGs (differentially expressed genes) shared in the two data sets. After filtering out the DEGs with low expression value [max (RPKM) > 1], we took the genes common to the two datasets. By analyzing the correlation between gene expression and citric acid content, we obtained 260 DEGs (the threshold value > 0.7). Hierarchical cluster analysis was performed using these 260 DEGs data by the pheatmap package of R with the clustering method set to complete and average linkage with Euclidean distance. Considering the hierarchical clustering result of all samples and the physiological data, the data were divided into two subclasses on the dimension of samples. The mean value and the ratio of the two subclasses were calculated for each candidate and used to do the entropy weight method analysis (Liu et al., 2019), and a relative ranking of all the 260 DEGs was obtained. Transcription factors whose expression patterns were positively correlated to the *CitALMT*’s were identified by the calculation of the Pearson Correlation Coefficient (PCC > 0.6).

### Organic Acid Measurement by GC–MS

Citrate concentration in fruits and transgenic callus was measured according to Li et al. (2017). Samples were ground in liquid nitrogen, and 1.4 ml chromatographic methanol was added to 0.1 g sample; after vortex for three times, the sample was placed at 70°C for 15 min and then centrifuged at 11,000 *g* for 10 min at 4°C. 0.75 ml trichloromethane and 1.5 ml purified water were added to the upper phase. After vortex fully for 3 times and centrifuging, an internal standard was a 100 μl aliquot of each sample with 20 μl ribitol (0.2 mg/ml). After vacuum drying the sample, adding 60 μl of 20 mg/ml methoxyamine hydrochloride dissolved in pyridine to the residue and incubate for 1.5 h at 37°C with vortex. 40 μl Bis (trimethylsilyl) trifluoroacetamide (1% trimethylchlorosilane) was added to the sample and incubated for another 30 min at 37°C. Each sample was added to the injection bottle and injected into a GC–MS fitted with a fused-silica capillary column (30 m × 0.25 mm internal diameter, 0.25 μm DB-5MS stationary phase) with a split ratio of 10:1. The injector temperature was 250°C and the helium carrier gas flow rate was 1.0 ml/min. The column temperature was maintained at 100°C for 1 min, increased to 185°C at a speed of 3°C/min, then increased to 250°C at a speed of 15°C/min and maintained for 2 min. The MS operating parameters: ionization voltage 70 eV, ion source temperature 230°C, and interface temperature 280°C.

### Gene Isolation and Sequence Analysis

The *CitALMT* gene and relevant transcription factors were isolated based on the citrus genome database,^1^ with the primers listed in Supplementary Table S1. The amino acid sequence alignment analysis with several reported *ALMT* genes in fruit was carried out by DNAMAN (V6.0.3.99).

### RNA Extraction and cDNA Synthesis

Total RNA was extracted according to our previous report (Li et al., 2017). The genomic DNA was degraded with RNase-free DNase I (Ambion), total of 1.0 μg RNA was used for cDNA synthesis. According to the manufacturer’s protocol, first-strand cDNA synthesis was initiated with GoScript™ Reverse Transcriptase (Promega) following the manufacturer’s protocol. The actin gene was used to adjust the concentration of the template. Three biological replicates were set for each sampling site.

### Gene Expression Analysis

Ssofast Eva Green Supermix Kit and CFX96 (Bio-RAD) were used for RT-qPCR. The PCR reaction mixture (total volume of 20 μl) consisted of 10 μl 2× real-time PCR mix (Bio-Rad), 1 μl for each primer (10 μM), 2 μl for diluted cDNA, and 6 μl for DEPC H_2_O. The initial step of the PCR program was to cycle 50 times at 95°C for 5 min, then at 95°C for 10 s, 60°C for 10 s, and 72°C for 15 s. At the end of each run, each gene was analyzed by melting curves. The actin gene was used as the internal control (Li et al., 2017). The 2^(−△Ct)^ method was used to analyze the expression levels of genes. All the primers used for real-time PCR were in Supplementary Table S2.

### Transient Overexpression and RNAi in Citrus Fruits

Full-length *CitALMT*, *CitMYB52,* and *CitbHLH2* were amplified with primers (listed in Supplementary Table S3) and inserted into the pGreen II 002962-SK vector (Hellens et al., 2005). Forward and reverse PCR-amplified cDNA fragments of *CitALMT*, *CitMYB52,* and *CitbHLH2* were inserted into the pHB vector driven by 2× CaMV 35S (Zhang et al., 2020). All the constructs were electroporated into *Agrobacterium tumefaciens* GV3101. Fruits with the same size at 150 DAFB (with relative high citrate concentration) and 180 DAFB (with relative low-citrate concentration) were selected for overexpression and RNAi assays, separately. *Agrobacterium*-mediated transient transformation in fruit was based on previous reports with some modifications (Li et al., 2017). Uniform sections from the fruit were selected and infiltrated with either empty vector (SK or PHB, as control) or *Agrobacterium* culture containing the target gene. After infiltration for 5 days, the infiltrated sections were sampled for citrate and expression analysis.

### Genetic Transformation and Identification

For citrus callus transformation, the full-length of *CitALMT* was cloned into downstream of the CaMV 35S promoter of a modified pCAMBIA1301 vector with primers (listed in Supplementary Table S3). A GUS reporter gene following the CaMV 35S promoter in the pCAMBIA1301 vector was used for positive screening. The recombinant vector was then introduced into *A. tumefaciens* strain EHA105.

Valencia orange embryogenic calluses in tissue culture were used for transformation. The recombinant plasmid was introduced into the wild-type calluses using an *Agrobacterium*-mediated method, as described by Li et al., (2002) with minor modification. After 3 days of dark co-culture, the calluses were transferred into the MT selective medium containing 400 mg/L cefotaxime and 50 mg/L hygromycin at 25°C in the dark. The proliferated calluses were subcultured at 4-week intervals on fresh MT selective medium.

Transgenic callus lines overexpressing *CitALMT* were identified by RT-qPCR and GUS staining. RT-qPCR and citrate concentration analysis were performed as described above. Three biological replicates were performed for each line.

### Gus (β-Glucuronidase) Staining Assay

Histochemical staining confirmed that GUS reporter gene co-transformed with *CitALMT*. The GUS staining assay was carried out using the GUS staining kit (Real-Times Biotechnology, China) according to the manufacturer’s protocol.

### Dual-Luciferase Assay

Dual-luciferase assays were performed as described in our previous report (Xu et al., 2014). The promoter of the *CitALMT* was amplified with the primers described in Supplementary Table S1 and inserted into the pGreen II 0800-LUC vector. Full-length transcripts of the transcription factors were inserted into the pGreen II 002962-SK vector (SK) with the primers described in Supplementary Table S1.

All the recombinant vectors were electroporated into *Agrobacterium tumefaciens* GV3101. Osmotic buffer for *Agrobacterium* cultures (10 mM MES, 10 mM MgCl_2_, 150 μM acetosyringone, pH 5.6) was prepared to a concentration (OD_600_ = 0.75). A mixed *Agrobacterium* culture (v/v, 10:1) of transcription factors and promoters was infiltrated into tobacco leaves using a needleless syringe. Fluorescence intensity of Luc and Ren after infiltration for 3 days was measured using a dual-luciferase assay reagent (Promega) in the GlomaXTM 96 microplate luminescence apparatus (Promega). Tobacco plants were grown in a growth chamber, with 16:8 h light:dark cycles. Results were calculated from at least three separate experiments, each with at least five biological replicates.

### Subcellular Localization Analysis

The full-length of *CitALMT*, *CitMYB52*, and *CitbHLH2* without termination codon were cloned into pCAMBIA1300-sGFP, the empty GFP vector was used as controls. By *Agrobacterium*-mediated infiltration (GV3101), the constructed GFP vectors were transiently expressed in transgenic *N. benthamiana* leaves (stably transformed with nuclear location signal, NLS-mCherry; Li et al., 2017). The 35S-*CitALMT*-GFP *Agrobacterium* culture was mixed with the RFP *Agrobacterium* culture (as a marker located on the tonoplast). A Zeiss LSM710NLO confocal laser scanning microscope was used to image the green fluorescent protein (GFP) fluorescence in tobacco leaves after infiltration for 2 days. Primers used for GFP construction are described in Supplementary Table S3.

### Electrophoretic Mobility Shift Assay

The full-length of *CitbHLH2* was inserted into pET-32a (Clontech) to express a CitbHLH2-His fusion protein. The recombinant vector was transformed into *E.coli* strain BL21 which was then incubated with LB medium at 37°C until OD_600_ = 0.5. 0.5 mM isopropyl β-D-1-thiogalactopyranoside (IPTG) was added to the medium and then incubated at 37°C for 3 h. The *E. coli* cells were collected with 20 mM Tris–HCl buffer (0.1 M NaCl, pH = 8.0) and ultrasonicated on ice at 200 W with on/off at 3 s/2 s cycle for 15 min, then centrifuged at 9,000 *g* for 20 min at 4°C. Subsequently, the Ni-NTA resin (Transgene) was added to the supernatant to combine His-tagged proteins at 4°C for 1 h, which were eluted with elution buffers containing gradient imidazole (30, 300, and 500 mM).

EMSA detection was performed using the LightShift Chemiluminescent EMSA kit (Thermo) according to the manufacturer’s instructions. The probes were labeled from the 3′ biotin terminal by HuaGene (Shanghai, China), annealed with complementary oligonucleotide, heated at 95°C for 5 min, and then gradually reduced to 25°C at a rate of 0.1°C s^−1^, and transformed into a double-stranded DNA probe. The probes used for EMSA were listed in Supplementary Table S4. The binding specificity was tested by mutant probe and competition with unlabeled probes (50, 200, and 500-fold unlabeled oligonucleotides).

### Yeast Two-Hybrid Assay

Yeast two-hybrid assays (Y2H) were performed using the MatchmakerTM gold yeast two-hybrid system (Clontech, United States) to analyze protein–protein interactions. The full-length of *CitMYB52* and *CitbHLH2* was separately subcloned into pGADT7AD vector and pGBKT7 BD vector (primers are listed in Supplementary Table S4). Meanwhile, pGBKT7-53 BD and pGBKT7-Lam BD separately combined with pGADT7-T AD was used as positive and negative control.

### Bimolecular Fluorescence Complementation Assay

The full-length of *CitMYB52* and *CitbHLH2* were cloned into the C- and N-terminus of YFP vectors, respectively (Sainsbury et al., 2009), using the primers listed in Supplementary Table S4. All the recombinant constructs were transiently expressed in transgenic *N. benthamiana* leaves (stably transformed with nuclear location signal, NLS-mCherry) through *Agrobacterium*-mediated infiltration (GV3101) based on previous reports (Li et al., 2017). Two days after infiltration, the YFP fluorescence imaging of tobacco leaves was performed using a Zeiss LSM710NLO confocal laser scanning microscope.

### Coimmunoprecipitation (CoIP) Assay

The coding sequences of *CitMYB52* and *CitbHLH2* without stop codon were cloned with primers (listed in Supplementary Table S4) and fused with 4myc tags and 3HA tags separately, then ligated into pCAMBIA1300-221 vector. Recombinant vectors were electroporated into *Agrobacterium tumefaciens* GV3101 and then transiently expressed in *N. benthamiana* leaves (Li et al., 2018). Two d after infiltration, *N. benthamiana* leaves were ground in liquid nitrogen and proteins were extracted with extraction buffer (20 mM HEPES-KOH, pH 7.5, 40 mM KCl, 1 mM EDTA, 0.5% Triton X-100, and 1× protease inhibitors; Roche) in the proportion of 3 ml/g tissue powder. After centrifugation at 20,000 *g* for 10 min, 100 μl of supernatant was retained as input, and 1 ml supernatant was incubated with anti-HA agarose (Sigma-Aldrich) at 4°C for 2 h. The proteins retained on the beads were then washed for three times with wash buffer (20 mM HEPES-KOH, pH 7.5, 40 mM KCl, and 1% Triton X-100), boiled in 2% SDS sample buffer for 10 min for elution. Western blotting gel electrophoresis was used for analysis using anti-myc or anti-HA antibodies (Sigma-Aldrich).

### Western Blotting

Eluents and inputs in Co-IP assay were separated on 10% SDS-PAGE gels and transferred to a PVDF membrane. The membrane was blocked with 5% skim milk at room temperature for 2 h. e. Antibodies for protein tags (anti-myc and anti-HA) were added into the TBST buffer (Tris-buffered saline plus Tween20) at a ratio of 1:2,000 and incubated at room temperature for 2 h. The membranes were rinsed with TBST buffer for three times, 10 min each. The anti-rabbit/anti-mouse horseradish peroxidase secondary antibody was added at a ratio of 1:10,000 and incubated at room temperature for 1 h. After washing three times with TBST buffer, the membranes were observed with chemiluminescence system.

### Statistical Analysis

All experiments in this study included at least three biological replicates, and all data represented as the mean ± standard error. DPS 7.05 (Zhejiang University, Hangzhou, China) was used to calculate the least significant difference (LSD_0.05_). Student’s *t*-test was used to calculate the statistical significance of the difference with a confidence level of 95.0% (^*^p < 0.05), 99.0% (^**^p < 0.01), or 99.9% (^***^p < 0.001). The figures were drawn using Origin 8.0 (Microcal Software Inc).

## Results

### Identification of the *CitALMT* Gene by Transcriptome Analysis

Using RNA-seq results from our previous reports (Lin et al., 2015a,b), we identified 260 DEGs that are highly correlated with citrate and performed hierarchical clustering (the threshold value > 0.7; Supplementary Figure S1A). The data were divided into two subclasses on the dimension of samples which correspond to two sample sets with relatively high acid and low acid periods. We combined the maximum expression values and the fold change values of the subclasses using the entropy weight method and obtained a relative ranking of all the 260 DEGs (Supplementary Dataset S1). An aluminum-activated malate transporter named CitALMT (Ciclev10019573m) was ranked first (Supplementary Figure S1B). In addition, 22 transcription factors whose expression patterns were positively correlated to the CitALMT’s were identified (correlation coefficient > 0.6; Supplementary Figure S1C).

### Amino Acid Sequence Analysis and Subcellular Localization of CitALMT

**Figure 1:**
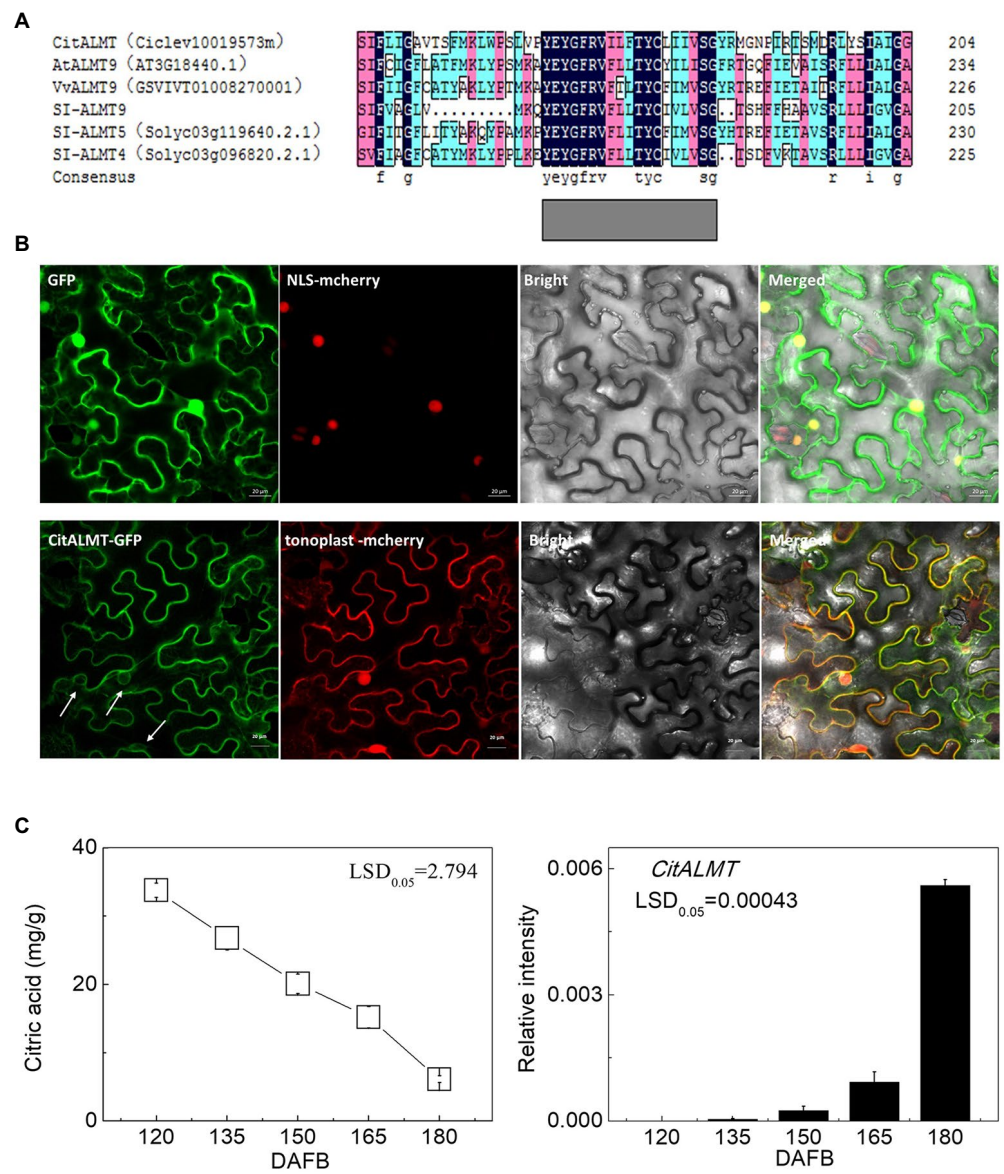
Identification of CitALMT. **(A)** The amino acid sequences analysis of CitALMT compared with some ALMTs reported previously. **(B)** Subcellular localization of CitALMT in tobacco leaves stably transformed with a red nuclear localization marker and agroinfiltrated with CitALMT-GFP and tonoplast-RFP marker. White arrows highlight co-localization of CitALMT with tonoplast marker. The empty vector control is on the top without tonoplast-RFP marker. The fluorescence was measured at 488 nm with a LSM780 microscope and photographed. Bars = 20 μm. **(C)** The citrate concentration and expression of the *CitALMT* genes in flesh of “Ponkan” fruits during the late stage of fruit development. DAFB, days after full blossom.

The amino acid sequence of *CitALMT* was compared to other ALMTs including *MA1* (MDP0000252114), *VvALMT9* (GSVIVT01008270001), *Sl-ALMT9*, *Sl-ALMT5* (Solyc03g119640.2.1), *Sl-ALMT4* (Solyc03g096820.2.1), *AtALMT9* (AT3G18440.1). The results indicated that *CitALMT* contains a conserved transmembrane domain TMD5 that may perform a transport function (Zhang et al., 2013; Figure 1A). The subcellular localization assay showed that CitALMT co-localized with tonoplast markers (Figure 1B).

### Correlation Between *CitALMT* Expression and Citrate Concentration in Citrus Fruit

The relationship between *CitALMT* expression and citrate concentration was further analyzed in multiple sets of citrus fruit at different developmental stages or after different treatments. During “Ponkan” fruit development, citrate concentration decreased continuously from 33.77 to 6.11 mg/g from 120 to 180 DAFB, while *CitALMT* transcripts increased significantly (Figure 1C).

In our previous research, citrate concentration was shown to decrease under hot air treatment (Li et al., 2021). Using these materials, we found the expression of *CitALMT* in hot air-treated “Ordinary Ponkan” was significantly higher than non-treated control. Moreover, “Ordinary Ponkan (OPK)” and its spontaneous early maturing mutant “Zaoshu Ponkan (ZPK)” were also used (Li et al., 2016). Compared with OPK, ZPK fruit had lower citrate concentrations and significantly higher *CitALMT* transcript level (Supplementary Figure S2).

### Transient Overexpression and RNAi Inhibition of *CitALMT* in Citrus Fruit and Stable Transformation of *CitALMT* in Callus

**Figure 2:**
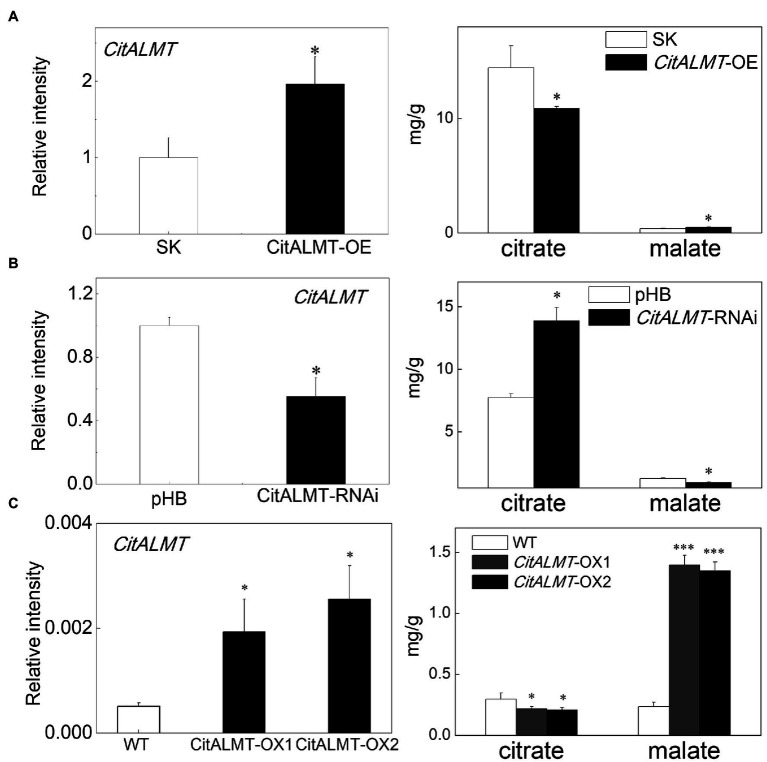
Gene function analysis of CitALMT. The transcript level of CitALMT (left) and organic acid concentration (right) were analyzed in citrus fruits and citrus callus. **(A)** Transient overexpression of *CitALMT* in citrus fruits. SK represents empty vector. **(B)** Transient RNAi of *CitALMT* in citrus fruits. pHB represents empty vector. **(C)** The analysis of transgenic *CitALMT* callus. Error bars indicate SE from three biological replicates. ^*^Significant differences (*p* < 0.05), ^***^Significant differences (*p* < 0.001).

To investigate the function of CitALMT, a rapid and efficient transient overexpression assay was performed in citrus fruit. The citrate concentration declined from 14.44 mg/g (in control fruits) to 10.87 mg/g (in *CitALMT*-OE fruits), while the malate concentration increased from the control value of 0.4 mg/g to 0.51 mg/g. The expression level of *CitALMT* was significantly increased in overexpressing fruits compared to the control (Figure 2A). For a better understanding of CitALMT’s role, we performed transient RNAi inhibition in citrus fruits. The results showed that the transcript level of *CitALMT* in *CitALMT*-RNAi fruit was reduced to 55% compared to the control fruit, while the citrate showed a significant increase from 7.73 mg/g in control to 13.89 mg/g and malate showed a significant decrease (Figure 2B).

In addition, stable transformation was performed in Valencia orange callus tissue. Two transgenic lines were obtained, as indicated by GUS staining (Supplementary Figure S4A) and RT-qPCR (Figure 2C). The expression of *CitALMT* in transgenic overexpressing lines OX-1 and OX-2 were 4-fold and 5-fold higher, respectively, than that of wild type. Citrate analysis showed that both transgenic lines had significantly lower citrate concentrations (0.21 mg/g and 0.20 mg/g, respectively) compared to wild-type callus (0.30 mg/g citrate). Meanwhile, the malate concentrations in transgenic callus were significantly enhanced approximately 5-fold (Figure 2C).

Citrate is degraded in the cytoplasm mainly through the GABA shunt. Thus, we analyzed the expression pattern of key genes (*CitAco3*-*CitIDH3*-*CitGAD4*) involved in the GABA shunt (Chen et al., 2012; Lin et al., 2016). The results indicated that the transcripts of these three genes in *CitALMT*-overexpressing fruits were significantly higher than in the control. In contrast, transcripts of these three genes in the GABA shunt were decreased in *CitALMT*-RNAi fruits (Supplementary Figure S3). RT-qPCR analysis indicated that both transgenic callus lines showed higher transcript levels of GABA shunt genes than wild type (Supplementary Figure S4B).

### *In vivo* Regulatory Effects of Transcription Factors on *CitALMT* Gene Promoter

**Figure 3:**
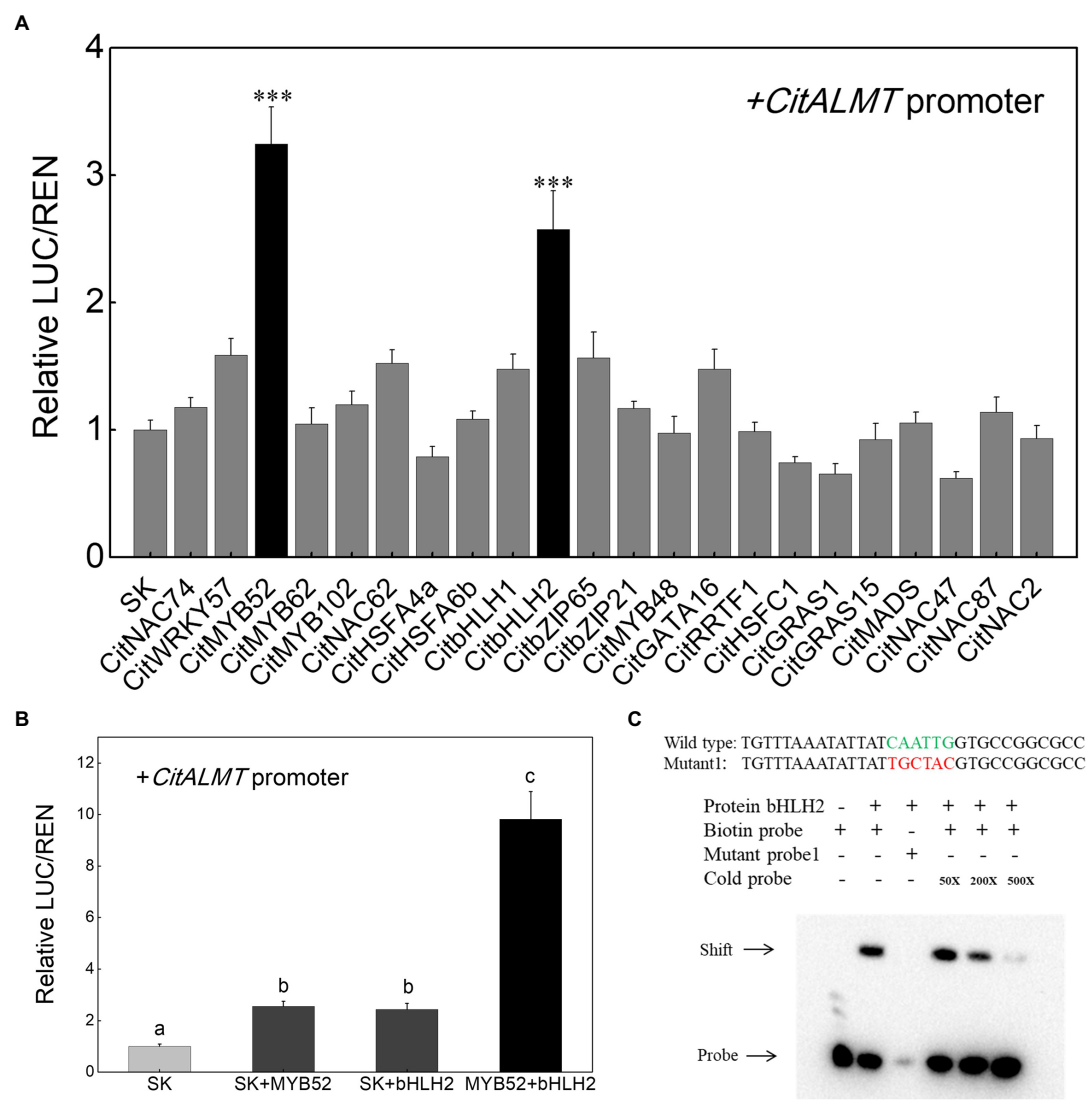
Interaction between transcription factors and *CitALMT*’s promoter. **(A)** *In vivo* interaction of transcription factors with the promoter of the *CitALMT* gene from “Ponkan” fruit. *In vivo* associations of the transcription factors and promoter were obtained by transient expression assays in tobacco leaves. The ratio of LUC/REN of the empty vector (SK) plus promoter was used as calibrator (set as 1). Error bars indicate SE from at least five replicates. ^***^Significant differences (*p* < 0.001). **(B)** Effect of the combination of CitMYB52 and CitbHLH2 on the *CitALMT*’ promoter. The ratio of LUC/REN of the empty vector (SK) plus promoter was used as calibrator (set as 1). Error bars indicate SEs from five biological replicates. Different letters above the columns represent significant differences (the combination effects were compared to two individual effects, *p* < 0.05). **(C)** Electrophoretic mobility shift assay (EMSA) of CitbHLH2 binding to the CitALMT promoter. Purified CitbHLH2 proteins and biotin-labeled DNA probe were mixed and analyzed on 6% native polyacrylamide gels. The presence (+) or absence (−) of specific probes is indicated. The concentration of the cold probe is shown; the biotinylated probe concentration was 1 nM.

The regulatory effects of the 22 identified transcription factors on *CitALMT* were tested using dual-luciferase assays and the results showed that in the presence of CitMYB52 or CitbHLH2, the transcriptional activity of the *CitALMT* promoter was significantly increased, about 3.2-fold and 2.6-fold, respectively, while the other tested transcription factors showed no significant effects (Figure 3A). Furthermore, we found that the combination of CitMYB52 and CitbHLH2 resulted in 9.8-fold induction of *CitALMT* promoter activity compared to CitMYB52 or CitbHLH2 alone (2.5-fold, 2.4-fold, respectively; Figure 3B).

### *In vitro* Interaction between CitbHLH2 and Promoter of *CitALMT*

Based on dual-luciferase assays (Figures 3A,B), the mechanism by which CitMYB52 and CitbHLH2 regulate the *CitALMT* promoter was further investigated. An EMSA assay showed that CitbHLH2 could physically bind to an E-box motif (CAATTG) in the *CitALMT* promoter. The specificity of the interaction was confirmed by mutated probes and cold probe competition experiments (Figure 3C). In contrast, CitMYB52 did not directly interact with the putative MYB-binding element on the promoter of *CitALMT* in this assay (Supplementary Figure S5). The promoter sequence and Cis-elements information of CitALMT are shown in Supplementary Table S5.

### Subcellular Localization and Transcript Levels of CitMYB52 and CitbHLH2

**Figure 4:**
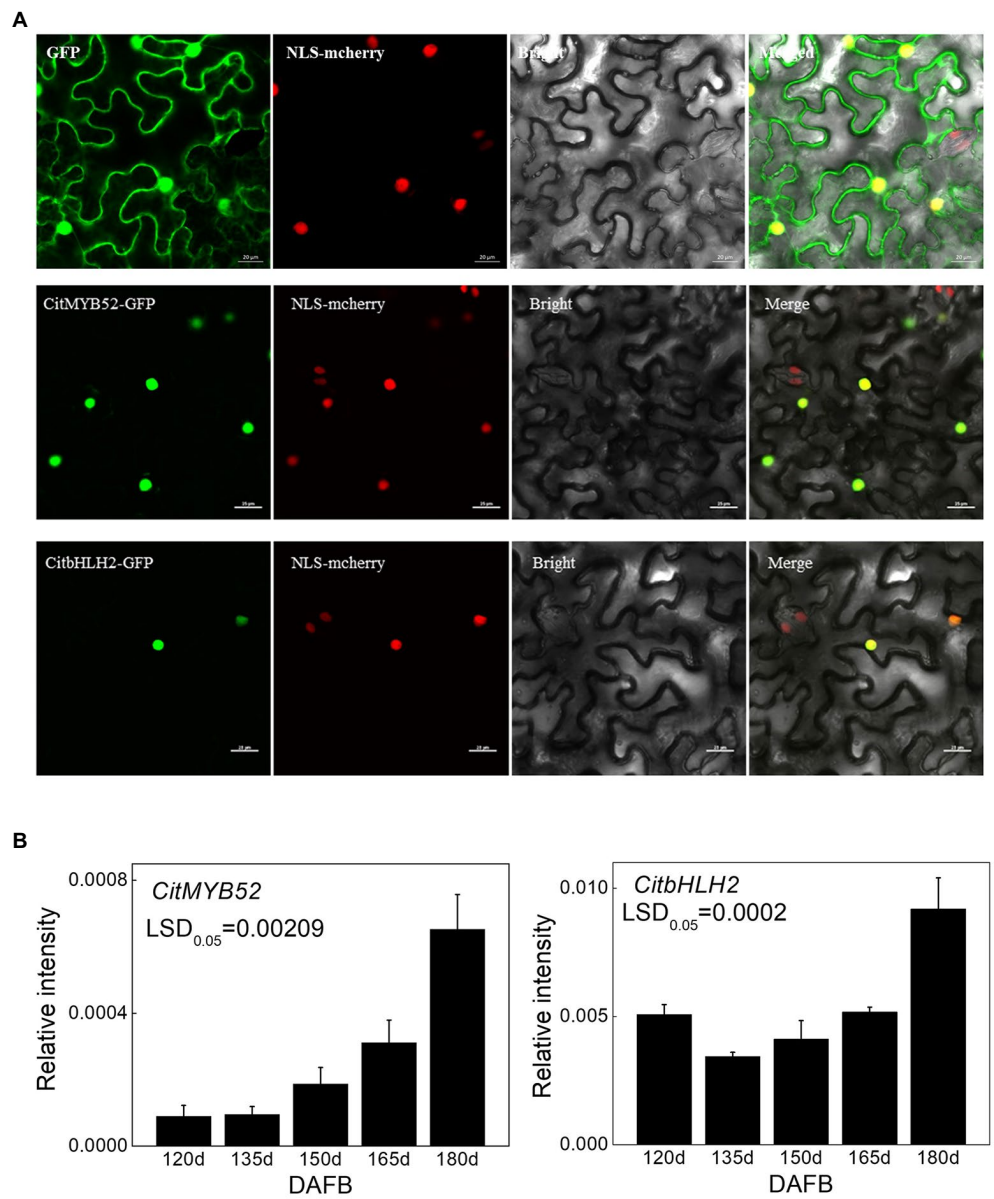
Characterization of CitMYB52 and CitbHLH2. **(A)** Subcellular localization analysis was performed in *N. benthamiana* leaves stably transformed with a red nuclear localization marker. GFP fluorescence of CitMYB52-GFP and CitbHLH2-GFP are indicated. The empty vector control is at the top. Bars = 25 μm. **(B)** Expression of the *CitMYB52* and *CitbHLH2* genes in the flesh of “Ponkan” fruits during the late stage of fruit development, DAFB, days after full blossom. Error bars represent SE (*n* = 3).

A subcellular localization assay was performed to visualize the locations of the two transcription factors and the results showed that CitMYB52 and CitbHLH2 were both located within the nucleus (Figure 4A).

Both *CitMYB52* and *CitbHLH2* exhibited a rising trend in expression which was negatively correlating with citrate concentration, excluding the 120 DAFB in *CitbHLH2* time point (Figure 4B).

### Protein–Protein Interaction Between CitMYB52 and CitbHLH2

**Figure 5:**
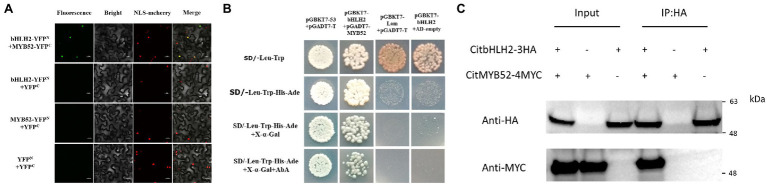
Protein–protein interaction between CitMYB52 and CitbHLH2. **(A)** *In vivo* interaction between CitMYB52 and CitbHLH2, determined using BiFC. N- and C-terminal fragments of YFP (indicated on the figure as YFPN and YFPC) were fused to the C terminus of CitbHLH2 and CitMYB52, respectively. The pairs of fusion proteins tested were CitbHLH2-YFPN+CitMYB52-YFPC. The other combinations were negative controls. Fluorescence of YFP represents protein–protein interaction. Bars = 50 μm. **(B)** Interaction between CitMYB52 and CitbHLH2 in yeast two-hybrid assays. Liquid cultures of double transformants were plated at OD600 = 0.01 dilutions on synthetic dropout selective media: (1) SD medium lacking Trp and Leu; (2) SD medium lacking Trp, Leu, His, and Ade; and (3) SD medium lacking Trp, Leu, His, and Ade, and supplemented with AbA. pGBKT7-p53 and pGADT7-T were used as positive controls, while pGBKT7-Lam and pGADT7-T were used as negative controls. **(C)** Interactions between CitMYB52 and CitbHLH2 measured by CoIP. Total protein extracts (Input) and protein complexes immunoprecipitated with anti-HA agarose (IP) were separated on gels and blotted. Anti-HA and anti-myc antibodies were used in Western blotting.

Based on the EMSA results, the relationship between CitMYB52 and CitbHLH2 was further studied. BiFC assays showed that all the negative controls (empty vector and a combination of one gene with empty vector), including YFP^N^/CitMYB52-YFP^C^, CitbHLH2-YFP^N^/YFP^C^, and YFP^N^/YFP^C^, produced no fluorescence signal. Co-expression of CitMYB52-YFP^C^ and CitbHLH2-YFP^N^, however, resulted in a positive signal in the nucleus (Figure 5A), indicating a protein–protein physical interaction between CitMYB52 and CitbHLH2. Furthermore, this interaction was verified by yeast two-hybrid assay and CoIP assay with HA and myc antibody (Figures 5B,C). Taken together, these results suggest that CitMYB52 and CitbHLH2 act cooperatively to increase *CitALMT* promoter activity.

### CitMYB52 Interacts With CitbHLH2 to Co-regulate Citrate Concentration

**Figure 6:**
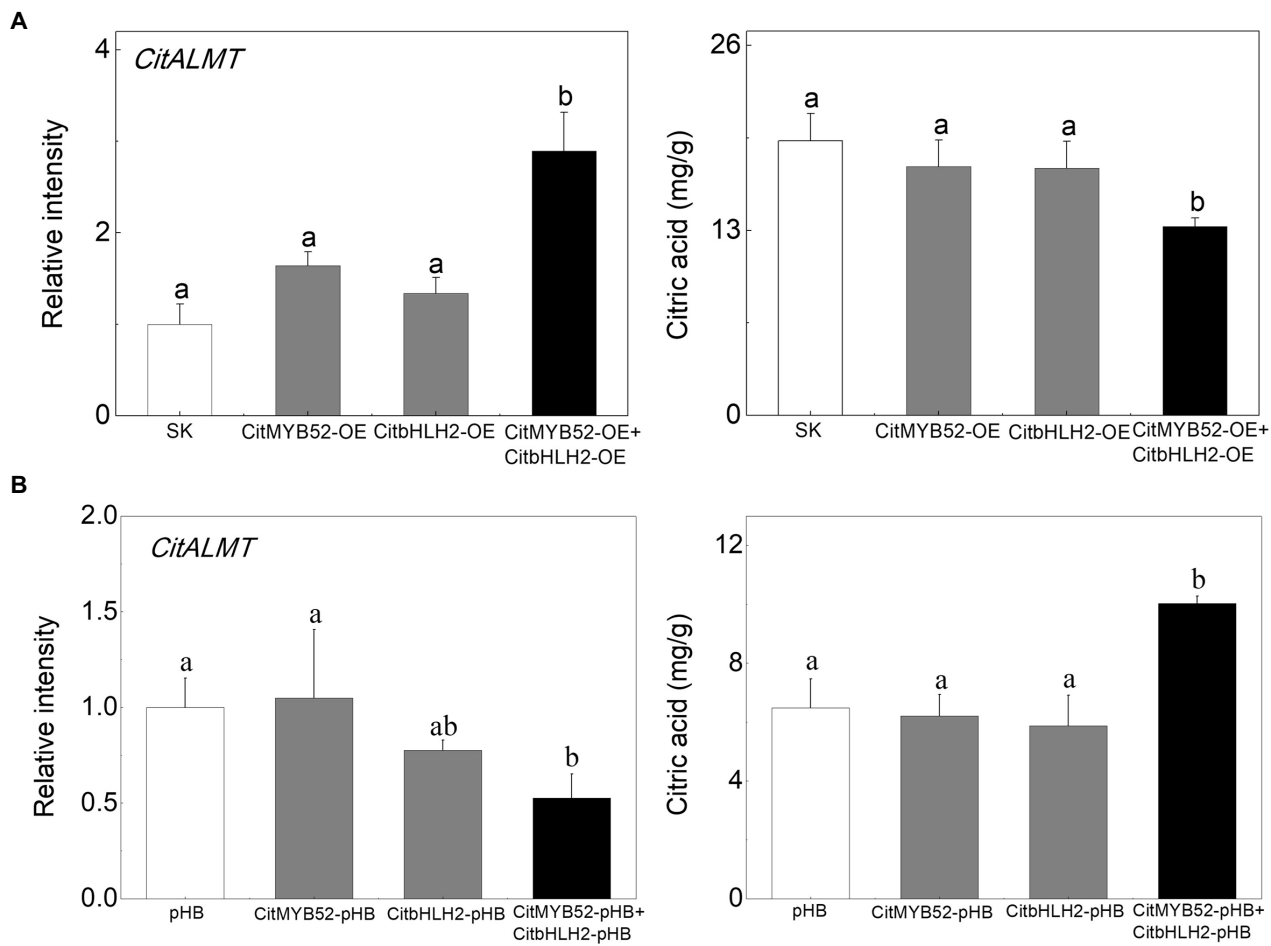
Effects on the citrate concentration of transient overexpression or RNAi inhibition of CitMYB52 and CitbHLH2. The transcript level of *CitALMT* (left) and citrate concentration (right) were analyzed in citrus fruits. **(A)** Transient overexpression of *CitMYB52* and *CitbHLH2* in citrus fruits. SK represents empty vector. **(B)** Transient RNAi of *CitMYB52* and *CitbHLH2* in citrus fruits. pHB represents empty vector. Error bars indicate SE from three biological replicates. Different letters above the columns represent significant differences (*p* < 0.05).

In order to investigate the effects of the two transcription factors on citrate, CitMYB52 and CitbHLH2 were introduced into citrus fruits by *Agrobacterium*-mediated transient transformation. The transcript levels of CitMYB52 and CitbHLH2 in overexpressing and RNAi fruits are indicated in Supplementary Figure S6 compared to the empty vector control. Transient overexpression of CitMYB52 and CitbHLH2 separately produced no difference in the citrate concentration in citrus fruits. However, the combined expression of CitMYB52 and CitbHLH2 significantly decreased the citrate concentration from 19.28 mg/g to 13.28 mg/g. In addition, the transcript abundance of *CitALMT* significantly increased 2.9-fold in *CitMYB52* and *CitbHLH2* dual overexpressing fruits (Figure 6A). Transient RNAi inhibition of both CitMYB52 and CitbHLH2 reduced the transcript level of *CitALMT* and increased the citrate concentration in citrus fruits (Figure 6B). These results indicated that these two transcription factors work together to influence citrate concentration by regulating the transcript level of *CitALMT*. In addition, the transcript levels of *Aco3*, *IDH3,* and *GAD4* were in general higher in dual overexpressing fruits and lower in dual RNAi fruits (Supplementary Figure S7).

## Discussion

### CitALMT Negatively Regulates Citrate Accumulation in Citrus

ALMT was originally characterized as a type of aluminum-activated malate transporter that is responsible for the exudation of malate in plant roots to chelate aluminum ions (Sasaki et al., 2004; Hoekenga et al., 2006). Subsequent research revealed that the physiological role of ALMT is not limited to the detoxification process of aluminum ions (Palmer et al., 2016; Sharma et al., 2016). In addition to malate, ALMTs may also mediate a bidirectional (i.e., efflux or influx) transport permeability and selectivity to both organic anions (i.e., citrate or tartrate) and inorganic anions (i.e., NO^3−^ or Cl^−^; Ligaba et al., 2012; De Angeli et al., 2013; Sasaki et al., 2016). For example, ZmALMT2 exhibits a high permeability to malate and citrate in maize (Ligaba et al., 2012). HvALMT1 can utilize malate, fumarate, or citrate as the substrate for the transport process in barley (Gruber et al., 2010). VvALMT9 is thought to mediate the transport process of both tartrate and malate, leading to the accumulation of malate and malate in grapes (De Angeli et al., 2013). In fruits, it is widely known that organic acid transporters may regulate the accumulation of various organic acids simultaneously (Huang et al., 2021). Citrate transport through the tonoplast may occur *via* malate channels in most fleshy fruits (Etienne et al., 2013; Huang et al., 2021). In tomato fruit, Overexpression of *SlTDT* increased the malate concentration significantly, while the citrate concentration is reduced, indicating that SlTDT may play a crucial role in the distribution of malate and citrate in tomato vacuoles (Liu et al., 2017). However, unlike malate, due to the lack of reports on citrate transporters, the role of ALMT on the accumulation of citrate has not received enough attention in fruits, although a genome-wide association study (GWAS) using 775 tomato accessions has been reported that Sl-ALMT9 on tomato chromosome 6 was significantly associated with both malic acid and citric acid (Zhao et al., 2019).

In our study, the transcriptomic analysis and expression profiles showed that *CitALMT* was negatively correlated with citrate concentration (Figure 1C; Supplementary Figures S1, S2), indicating that *CitALMT* may be correlated with the reduction of citrate. Because of the challenges associated with obtaining transgenic fruiting citrus plants, transient overexpression and RNAi assay in citrus fruits and transgenic callus were conducted to analyze the role of CitALMT. Overexpression of *CitALMT* in citrus callus and fruits could effectively promote the accumulation of malate while *CitALMT*-RNAi fruits exhibited a decreased malate concentration, which was in keeping with previous suggestions that ALMT could promote malate accumulation in fruits. Moreover, overexpressing or decreasing *CitALMT* transcript level significantly reduced or increased citrate concentration, respectively (Figure 2). These results indicated that CitALMT could negatively regulate citrate accumulation but promoted malate accumulation. We proposed that CitALMT functions as a channel protein that may affect the reallocation of citrate and malate in vacuoles.

In mature citrus fruits, most of the citric acid is stored in vacuoles. During the late stage of fruit development, citric acid is moved from the vacuole to the cytoplasm, where excess citrate is degraded by activating the GABA shunt genes (Cercós et al., 2006). Our results indicate that the transcript levels of GABA shunt genes were upregulated in the *CitALMT*-overexpressing fruits and callus transgenic lines, while downregulated in the *CitALMT*-RNAi fruits (Supplementary Figures S3, S4). These results raised a possibility that CitALMT, as a channel protein, may function to regulate the reallocation of citrate, which in turn affects the transcript levels of GABA shunt genes to regulate the degradation process of citric acid.

### CitMYB52 and CitbHLH2 Act as Transcriptional Activators of *CitALMT*

Transcription factors regulate the structural genes and transporters in plants. Overexpressing *SIAREB1* in tomato upregulated the expression of *mCS* and resulted in increased citrate and malate accumulation (Bastías et al., 2011); MdMYB1 was reported to be involved in malate accumulation by transcriptionally activating *MdVHA-B1* and *MdVHA-B2* in apple (Hu et al., 2016). In citrus, Noemi, a bHLH transcription factor, was essential for the production of flavonoid pigments and regulation of fruit acidity in citrus (Butelli et al., 2019). Our previous reports identified an ethylene response factor, CitERF13, which interacts with a vacuolar proton pump CitVHA-c4 to regulate citrate accumulation (Li et al., 2016); A heat shock transcription factor CitHsfA7 participates in citric acid degradation in citrus fruit *via* modulating *CitAco3* (Li et al., 2021).

In recent years, some transcription factors that show regulatory effects on *ALMT* have been characterized. For instance, AtSTOP1, a C2H2 type transcription factor, is crucial to malate excretion by activating *AtALMT1* expression in *Arabidopsis* (Iuchi et al., 2007). The apple MYB genes *MYB1/73* are reported to regulate malate accumulation by directly transcriptionally activating the expression of several proton pump genes and *MdALMT9* (Hu et al., 2016, 2017). A CALMODULIN-BINDING TRANSCRIPTION ACTIVATOR2 was identified as an activator of *AtALMT1* expression (Tokizawa et al., 2015), WRKY46 was shown to be a negative regulator of *ALMT1*, and the *wrky46* mutant showed increased malic acid secretion and decreased accumulation of aluminum in the root apices (Ding et al., 2013). In tomato, Sl-WRKY42 acts as a transcriptional repressor of *SlALMT9* (Ye et al., 2017). However, as with the research on the citrate transport process, the transcription factors involved in this process are also poorly studied. Here, the transcriptomic analysis identified 22 candidate transcription factors whose expression patterns were positively correlated to the CitALMT’s (Supplementary Figure S1C). Of these, CitMYB52 and CitbHLH2 showed significant regulatory activities on the *CitALMT* promoter (Figure 3A). This finding was further supported by associations between the expression of *CitMYB52* and *CitbHLH2* with *CitALMT*’s (Figure 4B). Moreover, EMSA analysis indicated that CitbHLH2 physically binds to E-box (CAATTG) within the *CitALMT* promoter (Figure 3C), which indicated the potential regulatory mechanism of the transcription factor by direct effects.

### CitMYB52 Interacted With CitbHLH2 Synergistically Regulating *CitALMT* and Negatively Regulating Citrate Concentration

We proposed that a protein–protein interaction between CitMYB52 and CitbHLH2 exists, based on the co-regulatory effect in dual-luciferase assays (Figure 3B). MYB and bHLH proteins control multiple pathways in numerous biological processes. The well-characterized functions involve the formation of ternary complexes of MYB-bHLH-WD40 that participate in the biosynthesis of flavonoid and anthocyanin, such as poplar MYB165 and bHLH131, kiwifruit AcMYB123 and AcbHLH42 (Ma et al., 2018; Wang et al., 2019). Recent research suggested the potential role of MYB and bHLH proteins on vacuolar acidification. In petunia petals, PH4-AN1-AN11 complex (a MBW complex) is important for vacuolar acidification by recruiting WRKY factor PH3 to regulate proton pump gene *CitPH5* (Verweij et al., 2016). In grape, VvWRKY26 enhances the activation effect of MBW complex on proton pump genes *VvPH1* and *VvPH5* (Amato et al., 2019). Mutations that disrupt the expression of homologous MYB, HLH, and/or WRKY transcription factors inhibited the expression of proton pump genes *CitPH1* and *CitPH5* to regulate citrus fruit acidity (Strazzer et al., 2019). This provides supporting evidence for the involvement of MYB-bHLH complex in regulating proton pump genes. Considering that the transport process of citric acid is also manipulated by channel proteins, however, the transcription complexes involved in this process are poorly identified.

Here, we identified two transcriptional activators CitMYB52 and CitbHLH2, which could both trans-activate *CitALMT* (Figure 3A). However, our transient overexpression assay suggested that overexpressing CitMYB52 or CitbHLH2 individually showed no significant effects on citrate concentration (Figure 6A). Y2H, BiFC, and CoIP assays confirmed a protein–protein interaction between CitMYB52 and CitbHLH2 exits (Figures 5A–C). Furthermore, this new CitMYB52-CitbHLH2 interaction synergistically trans-activated the promoter activity of *CitALMT* (Figures 3B, 6A) and significantly decreased citrate concentration (Figure 6A). Moreover, the RNAi assay verified that silencing of both *CitMYB52* and *CitbHLH2* suppressed the expression level of *CitALMT*, and thereby increased the citrate concentration in citrus fruits (Figure 6B). In addition, overexpression and silencing of both *CitMYB52* and *CitbHLH2* increased and reduced the expression levels of GABA shunt genes, respectively (Supplementary Figure S7), which corresponded to the transcript level of *CitALMT*. These results provided an example of MYB-bHLH mediated regulation of citrus fruit acidity by a carrier protein and important insights into the molecular mechanisms underlying acidity regulation in citrus fruits. Considering citrate is an important contributor to taste in citrus, the identification of this complex uncovers another instrumental step for organic acid accumulation.

## Conclusion

Citric acid plays an important role in the flavor, nutrition, and beverage quality of citrus fruits. Here, the transcriptomic analysis identified an aluminum-activated malate transporter (CitALMT) and 22 transcription factors that showed a high correlation to citrate accumulation. Using transient overexpression, RNAi, and stable transformation, CitALMT was confirmed as a novel transporter that negatively regulates citrate accumulation. Moreover, two transcription factors, named CitMYB52 and CitbHLH2 could interact with each other to co-regulate citrate accumulation by regulating the expression of *CitALMT.* These findings provide an important clue and new insights into the underlying regulatory mechanism of *CitALMT*, which opens the way to develop molecular breeding for improved citrus fruit acidity.

## Data Availability Statement

The original contributions presented in the study are included in the article/Supplementary Material, further inquiries can be directed to the corresponding author.

## Author Contributions

SjL and KC conceived the research plans and supervised the experiments. ScL and XL performed most of the experiments. CL and JS provided research material to ScL. BG and DW provided technical assistance to ScL. SjL, XY, and KC designed the experiments, analyzed the data, and wrote the article. DG revised the article with contributions of all the authors. All authors contributed to the article and approved the submitted version.

## Funding

This research was supported by National Key Research and Development Program (2016YFD0400101), the National Natural Science Foundation of China (31801591), and the Fundamental Research Funds for the Central Universities (2018FZA6010).

## Conflict of Interest

The authors declare that the research was conducted in the absence of any commercial or financial relationships that could be construed as a potential conflict of interest.

## Publisher’s Note

All claims expressed in this article are solely those of the authors and do not necessarily represent those of their affiliated organizations, or those of the publisher, the editors and the reviewers. Any product that may be evaluated in this article, or claim that may be made by its manufacturer, is not guaranteed or endorsed by the publisher.

## Footnotes

^1^ https://www.citrusgenomedb.org/
