# Supplementary material for: The Interaction Between CitMYB52 and CitbHLH2 Negatively Regulates Citrate Accumulation by Activating CitALMT in Citrus Fruit
Source: Front Plant Sci. 2022 Mar 21;13:848869. doi: 10.3389/fpls.2022.848869 (PMC8978962; doi:10.3389/fpls.2022.848869)
Supplement: Supplementary file 1 [file Table_1.DOCX]

**Table S1 Primers used for amplification of the promoter of *CitALMT* and SK construction**

| Primer name | Forward primer (5’ to 3’) | Reverse primer (5’ to 3’) | | |
| --- | --- | --- | --- | --- |
| *CitALMT*- LUC  *CitRRTF1*-SK  *CitGATA16*-SK  *CitMYB52*-SK  *CitHSTFA6B*-SK  *CitMYB62*-SK  *CitNAC62*-SK  *CitMYB102*-SK  *CitBHLH1*-SK  *CitWRKY57*-SK  *CitbZIP65*-SK  *CitHSTFC1*-SK  *CitMYB48*-SK  *CitNAC74*-SK  *CitbZIP21*-SK  *CitHSTFA4a*-SK  *CitMADS*-SK  *CitNAC87*-SK  *CitGRAS15*-SK  *CitNAC47*-SK  *CitGRAS1*-SK  *CitNAC2*-SK  *CitbHLH2*-SK | CTATAGGGCGAATTGGGTACCCCGGAATTAAGTATAAATTATG  AGCTCCACCGCGGTGGCGGCCGCATGCAAAGATCCTCAAAGCGACAG  AGCTCCACCGCGGTGGCGGCCGCATGGATGTGAAGACAAAAAGAA  AGCTCCACCGCGGTGGCGGCCGCATGGAGGATTCAGGAGCTGG  AGCTCCACCGCGGTGGCGGCCGCATGCATCCAACAGGTAGAGT  AGCTCCACCGCGGTGGCGGCCGCATGCACACAATGAGAGCAGC  AGCTCCACCGCGGTGGCGGCCGCATGGCAGTGTTGTCATTGAATT  AGCTCCACCGCGGTGGCGGCCGCATGGGAAGGCCACCAAGCTC  AGCTCCACCGCGGTGGCGGCCGCATGGGTTCTGAGTCTTCTGC  AGCTCCACCGCGGTGGCGGCCGCATGGATGATAGTAGCAAAGAG  AGCTCCACCGCGGTGGCGGCCGCATGGCTACTTCTACTAGTAAC  AGCTCCACCGCGGTGGCGGCCGCATGGAGCCCAACAACACCAAC  AGCTCCACCGCGGTGGCGGCCGCATGGACAGAGCAGGAGGACAT  AGCTCCACCGCGGTGGCGGCCGCATGGGGCTAAGAGATATTGG  AGCTCCACCGCGGTGGCGGCCGCATGGCGAGTCACAGAATTGGAG  AGCTCCACCGCGGTGGCGGCCGCATGGATGAATCACAGGGACAGG  TCCCCCGGGCTGCAGGAATTCATGGGTAGGGGAAAGATCGAGAT  TCCCCCGGGCTGCAGGAATTCATGGAGGAAGCCGTAGTTGATC  TCCCCCGGGCTGCAGGAATTCATGAGAGTTCCCCTAAAATCTC  TCCCCCGGGCTGCAGGAATTCATGGTTTGCATAAAGAACCCGG  TCCCCCGGGCTGCAGGAATTCATGGACTCTCGCCAGATTATTG  TCCCCCGGGCTGCAGGAATTCATGTGTGGCCTTGATTTCCCTG  TCCCCCGGGCTGCAGGAATTCATGGATTCAAGTACTAATCATAAT | | GTTTTTGGCGTCTTCCATGGCCAACCCTGTTTGCACACTCTGC  GATAAGCTTGATATCGAATTCTCATGAAGTAAGACCAGTGGCAGT  GATAAGCTTGATATCGAATTCTTAAGCATAAAGACAACCGT  GATAAGCTTGATATCGAATTCTCAAGAAGTGATACCGACAC  GATAAGCTTGATATCGAATTCTTACTTTGGACTCGAACTCAGG  GATAAGCTTGATATCGAATTCTTACTCCCTAAGCTGCCATATG  GATAAGCTTGATATCGAATTCTCAAAAATTAAGGCAAAGAC  GATAAGCTTGATATCGAATTCTTATACAAGTCCAATCGCAT  GATAAGCTTGATATCGAATTCTTATTTGTTTCTATCAGCAT  GATAAGCTTGATATCGAATTCTCATCTATTGCGCATCCCAG  GATAAGCTTGATATCGAATTCTCATTCCTGTCGAGGGCGAG  GATAAGCTTGATATCGAATTCCTAAAAACCGCCTCCTAGCAATG  GATAAGCTTGATATCGAATTCCTAGCCAGTTAAAGTTGCGG  GATAAGCTTGATATCGAATTCTCATAGGAAAACCATGCTGT  GATAAGCTTGATATCGAATTCTCAAAAGTTCGAGAAATGAT  GATAAGCTTGATATCGAATTCTCATCCAGCGGAAGTAAGGT  GGGCCCCCCCTCGAGGTCGACTCAGGCAAGGGTAAGATCACTG  GGGCCCCCCCTCGAGGTCGACTCAGTAGTCCCAAAAGCTATCC  GGGCCCCCCCTCGAGGTCGACCTAACACCTCCAAGCTGACGTG  GGGCCCCCCCTCGAGGTCGACTCATCCTTGAAACTGAAGATGT  GGGCCCCCCCTCGAGGTCGACTCAGTGCCAAGCAGAAGCAGAT  GGGCCCCCCCTCGAGGTCGACTCAATGAAGATACGCCATTTCC  GGGCCCCCCCTCGAGGTCGACTCAAACAATTTGATTCTCGACTG |  |

**Table S2 Primers for real-time quantitative PCR analysis**

| Gene | Forward primer (5’-3’) | Reverse primer (5’-3’) |
| --- | --- | --- |
| *CitALMT*  *CitMYB52*  *CitbHLH2*  *CitActin*  *CitAco3*  *CitIDH3*  *CitGAD4* | CAGGCACCCTACAATCTCCG  CTCGCCATCACACTTAAACT  AAGACTCTCAGCGACAACCG  CATCCCTCAGCACCTTCC  GCATGAGGCATGAGGATTC  TGTTTTTCTTTGAGGGGAATG  CGTCTCCGAAAGGAAAGCTA | GTCTATGCTGCCCTGGAGTC  AGAGCAAAATCAGTTAAATA  AGGGGGCCTTCTCCATTACT  CCAACCTTAGCACTTCTCC  TTGGCCAAAAGAAAAATGAA  GGAATTTCACAGGCAGCAAG  AAATAATCAAAATCGTCAACATGC |

**Table S3 Primers used in subcellular localization analysis, transient overexpression and RNAi analysis and genetic transformation analysis**

| Gene | Forward primer (5’-3’) | Reverse primer (5’-3’) |
| --- | --- | --- |
| *CitALMT*-GFP  *CitMYB52*-GFP  *CitbHLH2*-GFP  *CitMYB52*-SK  *CitbHLH2*-SK  *CitALMT* -SK  *CitALMT* -pSK1 (RNAi)  *CitALMT* -pSK2 (RNAi)  *CitMYB52-*pSK1 (RNAi)  *CitMYB52*-pSK2 (RNAi)  *CitbHLH2*-pSK1 (RNAi)  *CitbHLH2*-pSK2 (RNAi)  *CitALMT*-1301 | GTCGGTACCATGAATGGGAAAAAGGGTTCCGTTG  CTAGGTACCATGGAGGATTCAGGAGCTGGTTC  GCCGGTACCATGGATTCAAGTACTAATCATAAT  AGCTCCACCGCGGTGGCGGCCGCATGGAGGATTCAGGAGCTGG  TCCCCCGGGCTGCAGGAATTCATGGATTCAAGTACTAATCATAAT GCCGAATTCATGAATGGGAAAAAGGGTTCCGTTG  GCTCTCGAGTCTAGACAAAATGGGAGCCACCACAC  GGTGGATCCCAAAATGGGAGCCACCACAC  GCTCTCGAGTCTAGAAGAGGCTACTTGCAGCTCATC  GGTGGATCCAGAGGCTACTTGCAGCTCATC  GCTCTCGAGTCTAGATTGCCGCCTCATTATCCGAG  GGTGGATCCTTGCCGCCTCATTATCCGAG  AGAGAACACGCCCGGGGATCCATGAATGGGAAAAAGGGTTCCGTTG | CGCGTCGACAAAACTTGGTTGAACTAATAGACCCTC  GTTGTCGACAGAAGTGATACCGACACCGAGG  GTCGGATCCAACAATTTGATTCTCGACTGG  GATAAGCTTGATATCGAATTCTCAAGAAGTGATACCGACAC  GGGCCCCCCCTCGAGGTCGACTCAAACAATTTGATTCTCGACTG CGCGTCGACTCAAAAACTTGGTTGAACTAATAGACC  CACAAGCTTGCTTGGAATTTTCTGGCGGG  CACGAATTCGCTTGGAATTTTCTGGCGGG  CACAAGCTTGAACTTGTCACGATTGGGGC  CACGAATTCGAACTTGTCACGATTGGGGC  CACAAGCTTCGACTTCAGAGACCCGTGAC  CACGAATTCCGACTTCAGAGACCCGTGAC  CTTGCATGCCTGCAGGTCGACTCAAAAACTTGGTTGAACTAATAGAC |

**Table S4 Primers for EMSA, yeast two­hybrid, BiFC and CoIP assays**

| Gene | Forward primer (5’-3’) | Reverse primer (5’-3’) |
| --- | --- | --- |
| *CitMyb52*-pET32a  *CitbHLH2-*pET32a  *CitbHLH2*-Wild  Probe/ Cold probe (CitB5)  *CitbHLH2*-Mutant  Probe  CitM1-Wild  Probe  CitM2-Wild  Probe  CitM3-Wild  Probe  CitM4-Wild  Probe  CitM5-Wild  Probe  CitM6-Wild  Probe  CitB1-Wild  Probe  CitB2-Wild  Probe  CitB3-Wild  Probe  CitB4-Wild  Probe  CitB5-Wild  Probe  CitB6-Wild  Probe  CitB7-Wild  Probe  CitbHLH2-BD  CitMYB52-AD  CitbHLH2-YFP^N^  CitMYB52-YFP^C^  CitMYB52-CoIP  CitMYB52-myc  CitbHLH2-CoIP  CitbHLH2-HA | CGCGGATCCATGGAGGATTCAGGAGCTGGTTCT  GCCATGGCTGATATCGGATCCATGGATTCAAGTACTAATCAT  TTTAAATATTATCAATTGGTGCCGGCGCC  TGTTTAAATATTATTGCTACGTGCCGGCGCC  ACTTACAAGAATTTAGTTATTCATTAGGTA  GGAATTATAAGAATTTAGTTATTCATAAGATAAA  CAGATAATGAGTCTGTTATTTTTTTTTCGT  GAATAATAAGCACAATATCTCGTAAGAAAAT  CTTTCTTTCCCCAGTTGCACCTACAACTAAGCT  CTCCAGCTCCAGCCTCTCAGTTGAGCTCAAAAGC  CACATCACATGTTAATTACAACTTGTATATT  GGAAGAGTGTGCAGATGTTCATTTTCTATG  TTTTAACAACTTGCATTGCAACTCTCAGATGTTTA  GGTATAATTCCCATATGAGGAAAGTGGGAAG  TGTTTAAATATTATCAATTGGTGCCGGCGCC  TACAATATCCTACAAGTGCTATATATTTTCAC  CTTTCTTTCCCCAGTTGCACCTACAACTAAGCT  ATGGCCATGGAGGCCGAATTCATGGATTCAAGTACTAATCATAAT  GCCATGGAGGCCAGTGAATTCATGGAGGATTCAGGAGCTGGTTC  GCCTTAATTAATATGGATTCAAGTACTAATCATAAT  GCCTTAATTAATATGGAGGATTCAGGAGCTGGTTC  CACGGGGGACTCTAGAGGATCCATGGAGGATTCAGGAGCTGGTTC  GGTGTCGGTATCACTTCTGACAAGATGGAG  CACGGGGGACTCTAGAGGATCCATGGATTCAAGTACTAATCATAAT  CCAGTCGAGAATCAAATTGTTGTTACGTACATG | TATGTCGACAGAAGTGATACCGACACCGAGACC  TGCGGCCGCAAGCTTGTCGACAACAATTTGATTCTCGACTGGTT  GGCGCCGGCACCAATTGATAATATTTAAA  GGCGCCGGCACGTAGCAATAATATTTAAACA  TACCTAATGAATAACTAAATTCTTGTAAGT  TTTATCTTATGAATAACTAAATTCTTATAATTCC  ACGAAAAAAAAATAACAGACTCATTATCTG  ATTTTCTTACGAGATATTGTGCTTATTATTC  AGCTTAGTTGTAGGTGCAACTGGGGAAAGAAAG  GCTTTTGAGCTCAACTGAGAGGCTGGAGCTGGAG  AATATACAAGTTGTAATTAACATGTGATGTG  CATAGAAAATGAACATCTGCACACTCTTCC  TAAACATCTGAGAGTTGCAATGCAAGTTGTTAAAA  CTTCCCACTTTCCTCATATGGGAATTATACC  GGCGCCGGCACCAATTGATAATATTTAAACA  GTGAAAATATATAGCACTTGTAGGATATTGTA  AGCTTAGTTGTAGGTGCAACTGGGGAAAGAAAG  CCGCTGCAGGTCGACGGATCCTCAAACAATTTGATTCTCGACTG  CAGCTCGAGCTCGATGGATCCTCAAGAAGTGATACCGACACCGAG  ATAGGCGCGCCCAACAATTTGATTCTCGACTGG  ATAGGCGCGCCCAGAAGTGATACCGACACCGAGG  CTCCATCTTGTCAGAAGTGATACCGACACC  CGATCGGGGAAATTCGAGCTCTCTAGTCTAGCCC  CATGTACGTAACAACAATTTGATTCTCGACTGG  GATCGGGGAAATTCGAGCTCAGGCCCTTAAAGAC |

**Table S5 The promoter sequence and *Cis*-elements information of CitALMT**

| TAATTCCGGAATTAAGTATAAATTATGTAAATAAAATATGAAACTTGTAAATTAATATTAATTGTTAAATAAAATATAAATTATTTAAATAGAACGTAAGACTTATAAATGAAAAGTAAAATAAATAACTAACACATCACATGTTAATTACAACTTGTATATTAACATTACGTGTGAAATAAAACATAAATTATGTAAATGAAAGTAAAACAAGTAAATTAATATAAGAGTGATAAATTGATGCATTATTGATTAACAAAACAAAAACTTACAAGAATTTAGTTATTCATTAGGTAAAACTTGAAAATGTGCAGGTAAGGAAGAGTGTGCAGATGTTCATTTTCTATGTATACCGGATTTAGCGGTGTTCATTCTCTATATATCAGGTTTAGCTATCTATTTCTGGAATTATAAGAATTTAGTTATTCATAAGATAAAATTTGAGAATGTATGTAATTATTTTCTCCTTTTTATTGAAGTTTTATGTGTGACTTCCTGAAATTCTAATTACAGATAATGAGTCTGTTATTTTTTTTTCGTTATTGTTAATTTTCCACCTTAGCCACGCTTAATCAATCAAAAGCTTCATTCATCTTCAATTCTTCATCTCAAGTTATTCATTTTCTCTTGAAAATTGTTGATTTTTTTTTTTTTTTGGGGGGGGCCACGTTATTCCAGAATACATGCTTTACAAAATTAGTGGATGCATAATGAAAAGTAAATCGCAGAAGTTATCTGAGTGAAACACTTTTGACAGCAAAATGCTAAAAAATGGAGGGTTGATATAATTGAGTAATCCATCAAAATGCAAAATCCTACTAAAACGCACAGAAACCTTTATTTGATTCTATGTACGTGTCTAGGTAGATAAATATTGATGAGTTGCAAACATGCAAATTATTTATTAAAAAATAATTAAATACATGTAACAACATAAAGTAGGAAGAATAATGATATGAAGAATAATAAGCACAATATCTCGTAAGAAAATTTTGAGGAAAGCATGAGGTAGCCTAGGAAAGGCATATATTTCACGAAACATAATTAGATTCCTCCGACTAAATTAAATTTAGAGAGAAGAAAGCATTAATTTGTTGAGATATACACACACACACACACTTAATTCATTGAGAGTTATGGGTTGAAGATGAGGGAGCGAGCAAAAATCTTCAAAATTTTAACAACTTGCATTGCAACTCTCAGATGTTTAAATATTATCAATTGGTGCCGGCGCCATTCTCATCAGTCAAGGTATAATTCCCATATGAGGAAAGTGGGAAGCTTTTAAATCTCTTGTTTAATTTTTAACATTCTCCATTTTTTGTTCCCAATCTTCTTTTCCATTTTGGGTAAAACTCCGATCGAGGAATTGTTTAATATTGAAATGTAGAAATTAAATCACAAGCAAAGACGTTTATACATATCCTTGATCCGATAAAAACAAATTAATAAAGTCATCAACTAGAGTAGGTTATTACAGGCAATTAGTTTTGGACTTGCGTGTCTCAGAGCTTCAGCTTAACCTTAAAAAATAAAAATTAATAAGCATCGATCCTCTCTAACAATGAAATATTCTGAAGAGGTATACATAACACAAGTAATCGTGGTGTTGATTGATGCATTGTCATGCAATTTTGTTTTTTGACAAAATTTGTTTATTTATAGATAATATTCTAAATTCTCCATCCGTGCAACATTATTGAGGTTAAGCATCTTTATCCTGAGATTTTTAGTTCCATTTTCTCTTCTATTATTGCCGTATCATTCCCTCACTGTATATGATATATTGACTACACACGCTTTCTTTCCCCAGTTGCACCTACAACTAAGCTTACAATATCCTACAAGTGCTATATATTTTCACCTCAGCATTTCCTCCAGCTCCAGCCTCTCAGTTGAGCTCAAAAGCAGTTTATTTTAATTTCTAAAACAGCAATACGGGGAACTTGAAGCAGAGTGTGCAAACAGGGTTGGAAAATTTTATAGTACTTCATTGGAGA |
| --- |

**Note:** The underline represents the primer sequence used for EMSA assay. The yellow highlights represent the putative MYB-binding core elements, the green highlights represent the putative core E-box motif and the grey highlights represent the core elements both in MYB-binding motif and E-box motif.
