# Supplementary material for: The Interaction Between CitMYB52 and CitbHLH2 Negatively Regulates Citrate Accumulation by Activating CitALMT in Citrus Fruit
Source: Front Plant Sci. 2022 Mar 21;13:848869. doi: 10.3389/fpls.2022.848869 (PMC8978962; doi:10.3389/fpls.2022.848869)
Supplement: Supplementary file 2 [file Table_2.DOCX]

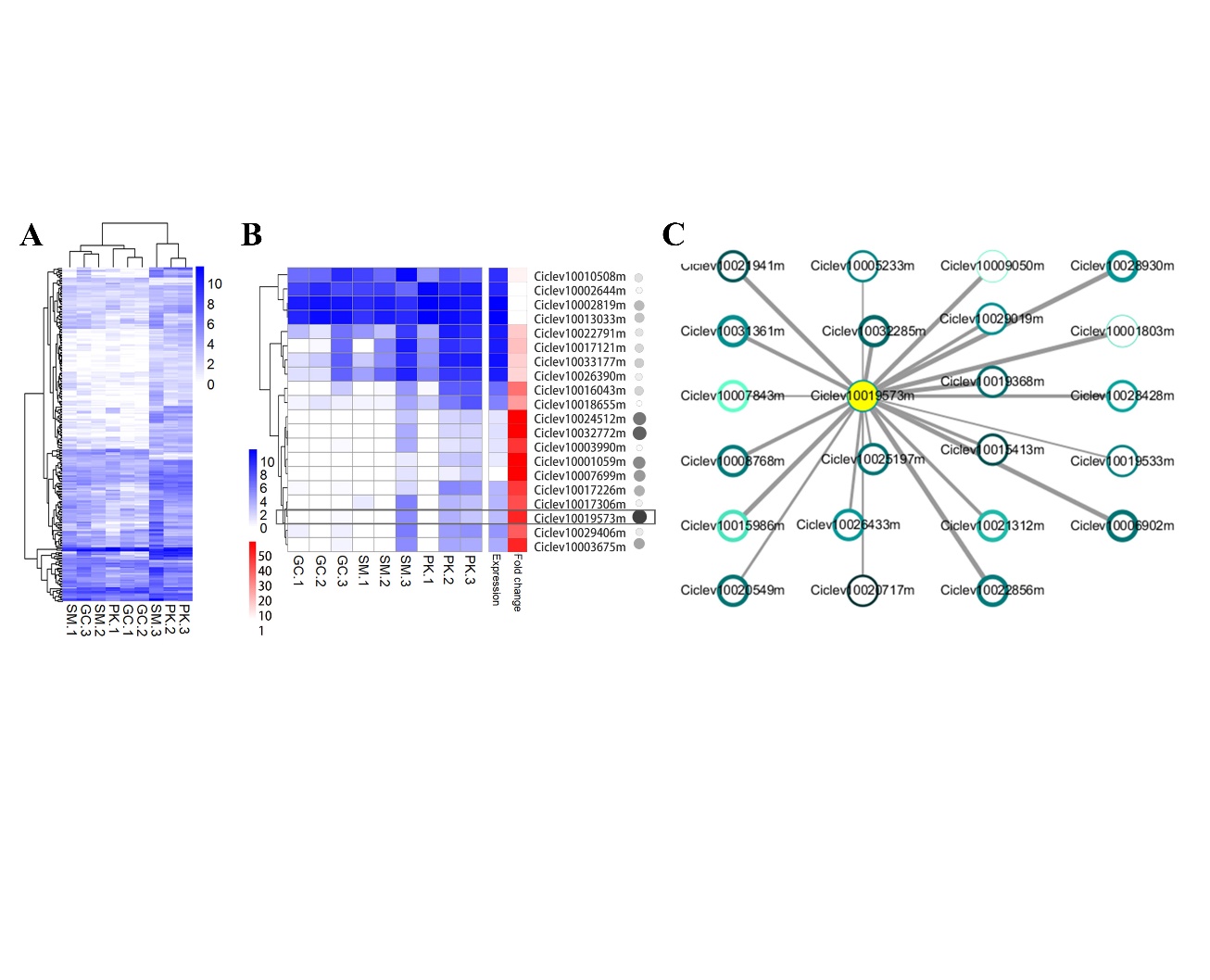


**Figure S1. Transcriptome analysis of differentially expressed genes (DEGs) at different developmental fruits stages of** **‘Gaocheng’, ‘Satsuma mandarin’ and ‘Ponkan’.** (A) Hierarchical clustering of all 260 differentially expressed genes. SM1, 2, 3 represent 60, 90, 180 DAFB in ‘Satsuma mandarin’; GC1, 2, 3 represent 75, 105, 195 DAFB in ‘Gaocheng’; PK1, 2, 3 represent 120, 195, 205 DAFB in ‘Ponkan’. (B) The expression details of top 20 DEGs. The dots represent the sorting. The larger the dot (the darker the color is), the higher the ranking. (C) 22 candidate TFs with expression patterns highly correlated with the *CitALMT*’s (Ciclev10019573m). The width of the edge and the color, width of the node represents the correlation coefficient, the expression level of genes and the expression fold change, respectively.


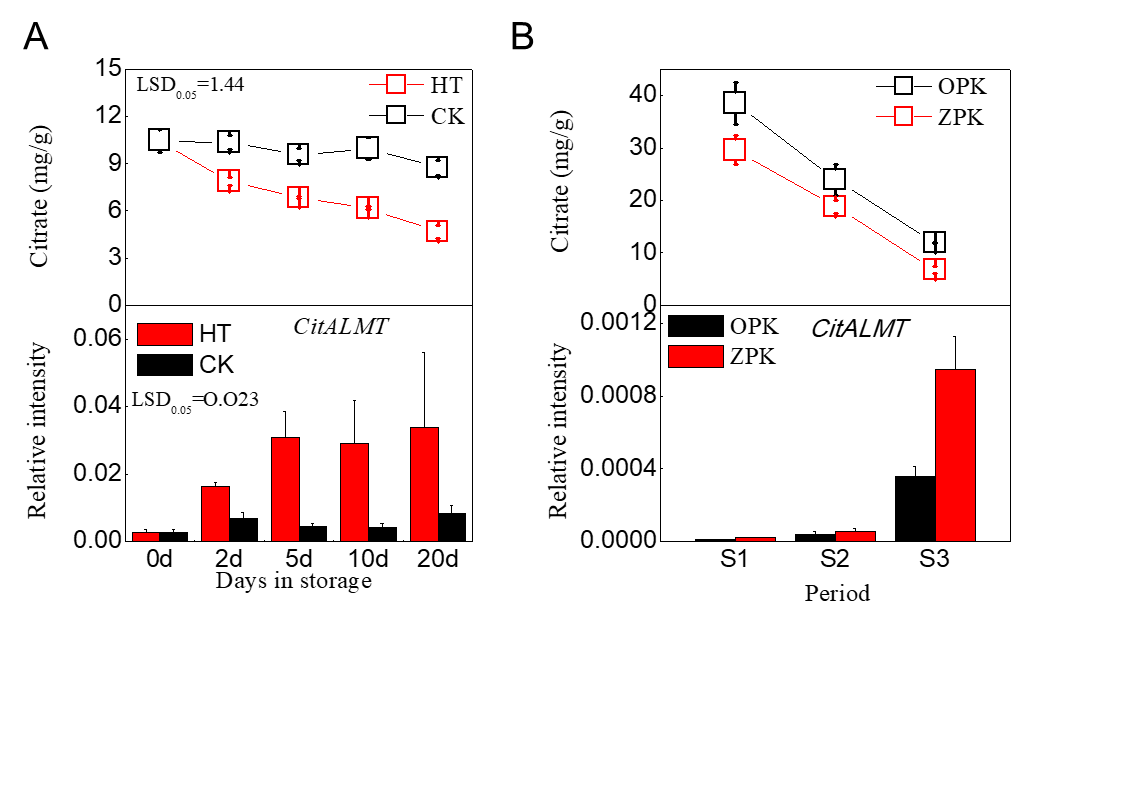


**Figure S2.** (A) The citrate concentration and expression of the *CitALMT* gene in ‘Ponkan’ fruits in response to hot air treatment. HT represents hot-air treatment; CK represents control. (B) The citrate concentration and expression of the *CitALMT* genes in the flesh of different ‘Ponkan’ fruits (‘Ordinary Ponkan (OPK)’ and ‘Zaoshu Ponkan (ZPK)’) during fruit development. S1、S2、S3 represent 120、150、180 DAFB respectively. Error bars represent SEs from three biological replicates.


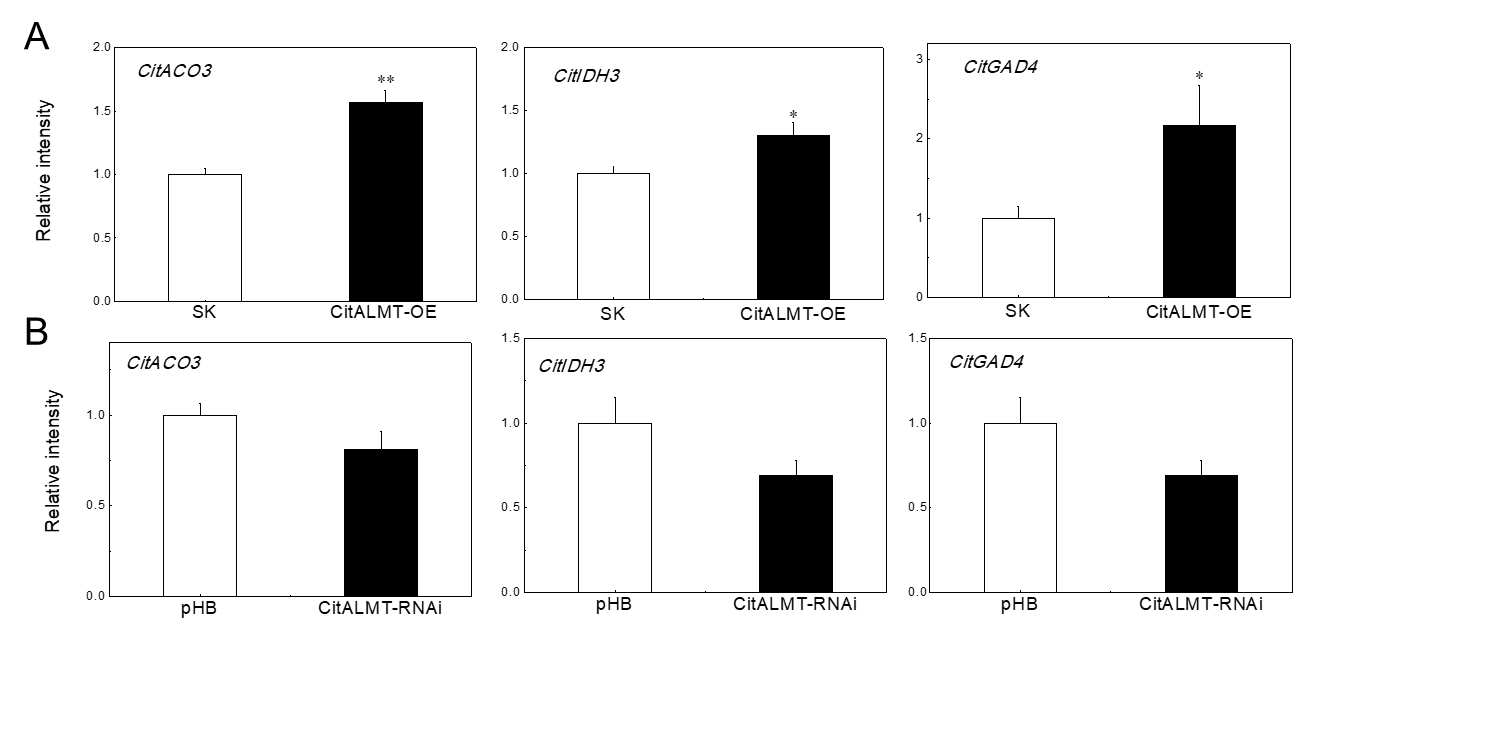


**Figure S3.** (A) The expression of the genes in GABA shunt in *CitALMT* over-expressed and control. (B) The expression of the genes in GABA shunt in RNAi fruits and control. Error bars indicate SE from three biological replicates. *Significant differences (P<0.05). **Significant differences (P<0.01).


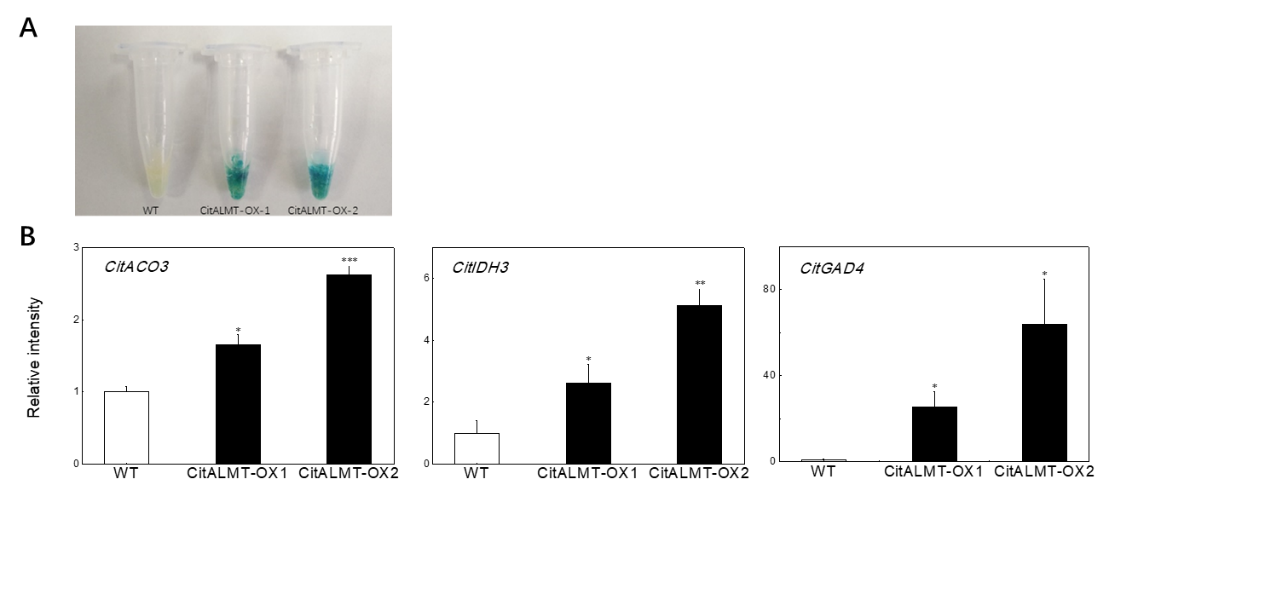


**Figure S4.** (A) Gus staining identifying of transgenic CitALMT callus. (B) the expression of the genes in GABA shunt in CitALMT transgenic citrus callus. Error bars indicate SE from three biological replicates. *Significant differences (P<0.05). **Significant differences (P<0.01). ***Significant differences (P<0.001).

**
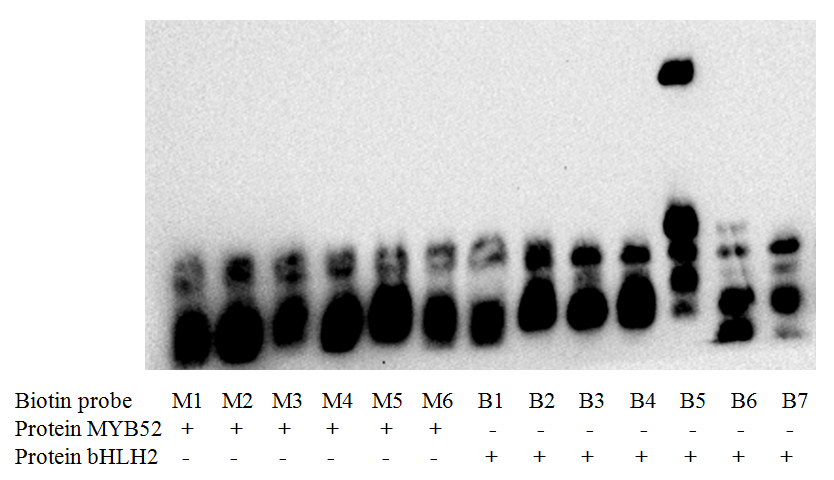
**

**Figure S5.** Electrophoretic mobility shift assay (EMSA) of CitMYB52 and CitbHLH2 binding to the *CitALMT* promoter. Purified CitMYB52 and CitbHLH2 proteins and putative TFs-binding biotin-labeled DNA probes were mixed and analyzed on 6% native polyacrylamide gels. The presence (+) or absence (-) of specific probes is indicated. The biotinylated probe concentration was 1 nM.


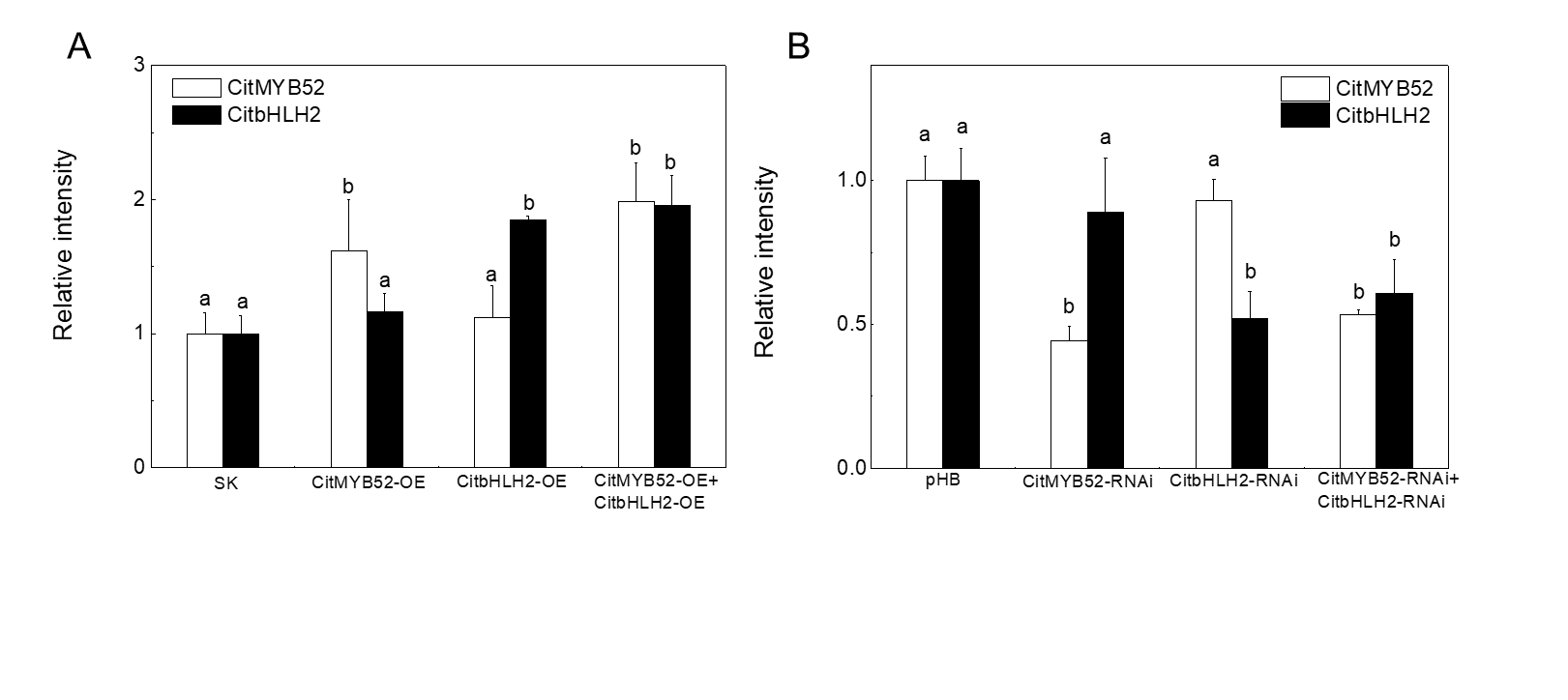


**Figure S6.** The expression of CitMYB52 and CitbHLLH2 in TFs-overexpressed (A) and RNAi (B) fruits. Error bars indicate SE from three biological replicates. Different letters above the columns represent significant differences (P<0.05).

**
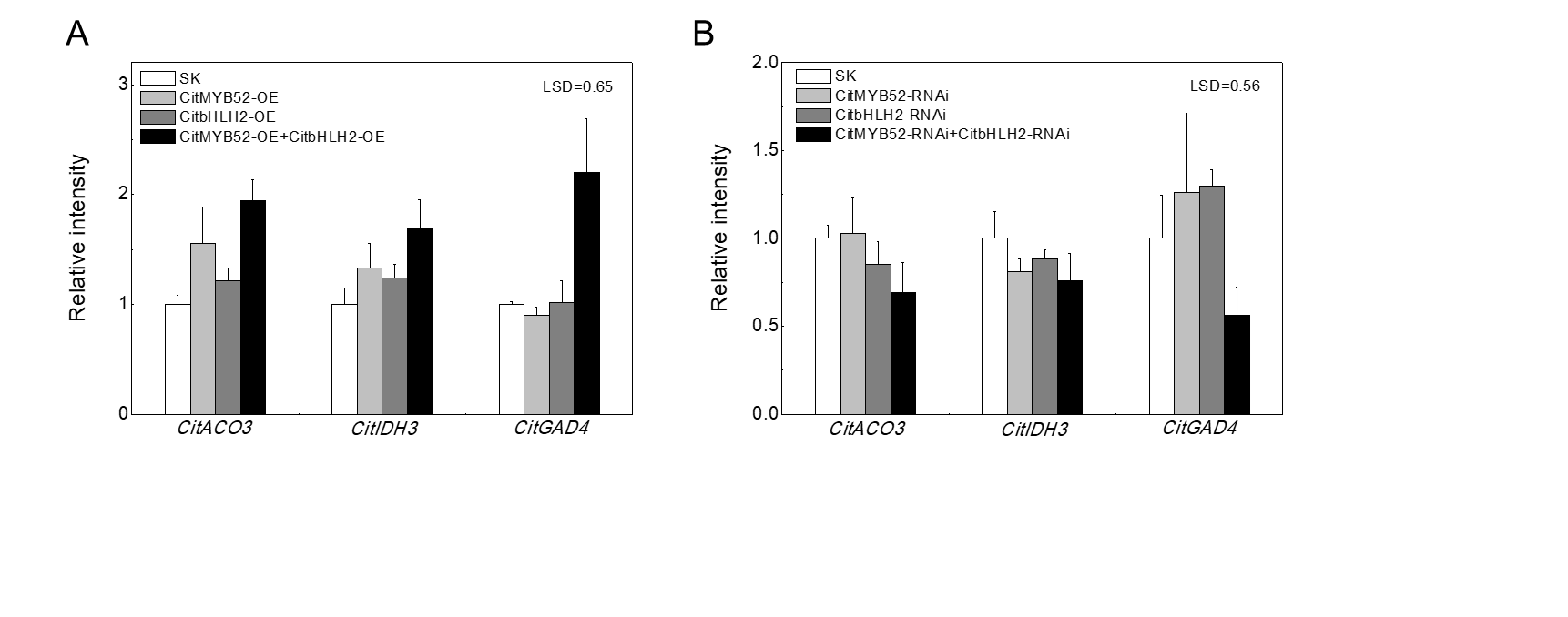
**

**Figure S7.** The expression of the GABA shunt genes in TFs-overexpressing (A) and RNAi (B) fruits. Error bars indicate SE from three biological replicates.
